
JOURNAL INFORMATION
==============================
NLM Title Abbreviation: South Med Rec
NLM Journal ID: 0027317

The Southern Medical Record

Publisher: Biomedical Journal Digitization Project

ARTICLE INFORMATION
==============================
PMCID: PMC9089669
Article version: 1
Subjects: Editorial and Miscellaneous

Obituary

Print publication date: 1879 Oct 20
Volume: 9
Issue: 10
First page: 400
Last page: 400
License: This work is free of known copyright restrictions. For more information, please see PMC Back Issue Digitization.
License URL: https://creativecommons.org/publicdomain/mark/1.0/

==============================

LARYNGEAL TUMORS AND TUBERCULOUS LARYNGITIS: By E. Fletcher Ingals, A. M., M. D.; Lecturer on diseases of the chest and physical diagnosis and on laryngotomy, in the Post Graduate Course, Rush Medical College. Reprinted from the Chicago Medical Journal and Examiner, for July, 1879.

THE RADICAL CURE OF HERNIA BY THE ANTISEPTIC USE OF THE CARBOLIZED CATGUT LIGATURE: By Henry O. Marcy, A. M., M. D., Cambridge, Mass ; member of the Massachusetts Medical Society, American Medical Association; Cambridge Medical Improvement Society; late Surgeon United States Army, etc.

THE TREATMENT OF EPITHELIOMA OF THE CERVIX UTERI: By J. Marion Sims, M. D.; Founder of the Woman's Hospital of the State of New York, and formerly Surgeon to the same; ex-President of the American Medical Association, etc., etc.

A CLINICAL TREATISE ON DISEASES OF THE NERVOUS SYSTEM: By M. Rosenthal, Professor of diseases of the nervous system, with a preface by Professor Charcot. Translated from the author's revised and enlarged edition by L. Putzel, M. D., visiting Physician for nervous diseases Randall's Island Hospital; Physician to the class for nervous diseases, Bellevue Hospital out door department., etc., etc.—Vol. II., New York: Wm. Wood & Co., 27 Great Jones street. 1879.

This is the second volume of this valuable treatise, the first of which we noticed in a previous number of this journal. It is an admirable work, replete with instruction and interest on a department of which less is known than, perhaps, any other branch of medical science. It is 277 octavo pages, neatly gotton up and illustrated.

### *OBITUARY.*

DIED, in Hopkinsville, Ky,, September 30th, 1879, Rev. Gideon B. Perry, D.D., L.L.D.

Dr. Perry was an eminent divine, and received also in addition to the degrees named, that of M.D., in Jefferson Medical College, Philadelphia. Truly a great and good man is gone!

RECEIPTED.—1879: L. G. Hardman; J. J. Grace; E. N. Cushing; J. T. Sego; J. W. Huff; A. F. Sanders; J. R. Wilson; A. J. Eilis; Gillispie & Payne; S. L. Lockwood; G. B. Battle'; A. L. Barry; A. J. Sewell; W. S. Posey; R. J. McMullen; J. W. Bennett; Geo. L. Mills; J. W. Buddeke.

### SPECIAL NOTICES.

WM. M. WARNER & Co.—This staunch and reliable house still maintains the high reputation for reliability and their goods and strict business integrity for which they have been so long characterized. Their sugar-coated pills are specially popular, not only in this country, but in European markets.

**Lactated Pepsine.**--This preparation is composed of the following powerful digestive principles, of which each dose of five grains contains: Pepsine (pure) 1 grain (equal to 5 grains of saccharated pepsine); Pancreatine, ½ grain; Lactic Acid, ½ grain; Maltose, ¼ grain; Diastase. 1-15 grain; Hydrochloric Acid, 1-10 grain. Doses 1 to 5 grains. Lactated Pepsine will be found of great value in dyspepsia, all phase of indigestion, malnutrition, diarrhœa in infants, chronic diarrhœa, cholera infantum, constipation, and vomiting in pregnancy.
Be particular to specify "PARKE, DAVIS & CO.'S LACTATED PEPSINE."

**Highest Prize Po t Wine.**--The best wine in the country that took the highest premium at the Centennial, is Speer's Port Grape Wine, which has become so celebrated. This wine and his P J brandy are now being used by physicians everywhere, who rely upon them as being the purest and best sold by Druggists. For sale by Druggisis.
